# The risk of long-term opioid use among immigrants: a national registry-linkage study

**DOI:** 10.1177/14034948241266744

**Published:** 2024-08-22

**Authors:** Håkon H. Nestvold, Svetlana Skurtveit, Aleksi Hamina, Vidar Hjellvik, Ingvild Odsbu

**Affiliations:** 1 Norwegian Centre for Addiction Research (SERAF), Institute of Clinical Medicine, University of Oslo, Oslo, Norway; 2 Department of Chronic Diseases, Division of Mental and Physical Health, the Norwegian Institute of Public Health, Oslo, Norway; 3 Department of Forensic Psychiatry, Niuvanniemi Hospital, Kuopio, Finland

**Keywords:** Nested case–control study, chronic pain, prescription drugs, long-term opioid use, immigration, socioeconomic status, analgesic, clinical relevance, healthcare utilisation, registries

## Abstract

**Aims:**

We aimed to investigate the association between being an immigrant and long-term prescription opioid use in Norway in 2010–2019.

**Methods:**

Nested case–control study. The cases were all persons 18 years of age or older with long-term opioid use – that is, the use of prescription opioids longer than 3 months (*N*=215,642). Cases were matched to four controls who filled at least one opioid prescription, but never developed long-term opioid use in the study period (*N*=862,568) on sex, age and year of starting long-term/short-term opioid use. Being an immigrant was defined as being born outside of Norway to two foreign-born parents and four foreign-born grandparents. Adjusting for socioeconomic variables and clinical confounders, analyses were stratified on three age groups (18-44 years, 45-67 years and ⩾68 years).

**Results:**

For the youngest age group, being an immigrant was inversely associated with long-term opioid use (adjusted odds ratio 0.75; 95% confidence interval [0.72–0.77]) compared with being native-born people. For this age group, the odds ratio differed between people born in Africa (0.56 [0.52–0.62]), Central or South America (0.70 [0.62–0.79]), Europe outside the European Union (EU) (0.71 [0.65–0.77]), Asia including Turkey (0.80 [0.77–0.84]) and EU/European Economic Area (EEA) (0.81 [0.77–0.85]). For the middle age group, increased odds were found for immigrants versus natives (1.05 [1.02–1.08]) in particular for those born in North America (1.26 [1.13–1.40]) and the EU/EEA (1.13 [1.09–1.18]). There was no association in the oldest group.

**Conclusions:**

Compared with native-born people, immigrants had lower odds of long-term opioid use among younger adults, higher odds among middle-aged and similar odds among older adults.

## Background

Opioid analgesics are often prescribed for postoperative pain, acute pain and cancer pain. They are also used to treat chronic pain but have less benefits and more potential for harm and addiction when prescribed for long periods [1, 2]. Socioeconomic factors are assumed to play a major role in a plethora of health issues, including the development of addiction and low socioeconomic status, and have been associated with an increased risk of opioid overdose, opioid use disorder and long-term opioid use in previous studies [1, 3–5]. Less is known about the risk of being prescribed opioids long-term among immigrants compared with native-born people, and the role socioeconomic status plays in the development of long-term opioid use in conjunction with health status among immigrants.

With major increases in global migration over the past decade [6, 7], immigrant health is a public health issue of growing importance for many countries. In Norway, the number of immigrants has tripled since the year 2000, which will lead to a larger proportion of immigrants in an aging population [8].

Immigrants are likely to differ in socioeconomic status from native-born people [4, 9]. Immigrants tend to have lower income, lower quality housing options and fewer employment options, even if their educational level is equal or higher compared with native-born people. However, immigrants tend to report better overall physical health than native-born people, except on somatic pain-related conditions [10, 11]. According to the Norwegian survey on living conditions in 2016, immigrants reported higher levels of several types of chronic pain, such as neuropathic pain, back pain and arthrosis, when compared with people born in Norway [12]. Similarly, a large Swedish registry study found that immigrants had a higher risk of chronic pain, widespread pain and severe pain when compared with the general population [13].

The prevalence of self-reported mental health issues among Norwegian immigrants is greatly dependent on their country of origin [11]. However, it has been shown that immigrants on average have a higher prevalence of mental health issues than native-born people, with anxiety and depression-related symptoms being most common [11]. Mental health issues are also more prevalent among immigrants with low education and unemployment, possibly due to economic problems and the stress of adapting to a new culture. Refugees and immigrants from conflict areas have a higher prevalence of mental health issues, but not necessarily a corresponding increased risk of substance use disorders [11]. Mental health disorders have been linked to long-term opioid use, opioid use disorder and fatal overdoses in previous studies [3, 5, 14, 15].

Immigration may have a protective effect for many types of substance use disorders, from alcohol to prescription opioid and illicit opioid use disorders [16–18]. Immigrants also seem to use fewer analgesics [11, 19, 20]. Possible reasons may be cultural differences in medication use, problems with the language and information access, perceptions of discrimination and financial issues [21, 22]. It has been shown that Norwegian immigrants use the healthcare system less than native-born people and receive fewer prescription drugs, with large variations between regions of birth [22–24]. A previous study from Norway showed that middle-aged people with immigrant background had lower odds of persistent opioid use when adjusting for socioeconomic factors, but without adjusting for clinical covariates [4]. To our knowledge, no previous study has investigated the interplay between immigration and the risk of developing long-term opioid use while considering both clinical and socioeconomic factors.

We aimed to investigate the association between immigration and the development of long-term opioid use in Norway during 2010–2019.

## Methods

### Study design and data sources

This is a nested case–control study based on data from several national health and population registers in Norway. Data from the registers were linked by each person’s unique personal identification number, which is assigned to everyone living in Norway for more than 6 months. See Hamina et al. [25] for a further description of the data linkage.

Data on filled prescriptions between 2010–2019 were retrieved from the Norwegian Prescription Database (NorPD) which contains information on all drugs dispensed from Norwegian pharmacies [25]. For each dispensing, we received information on sex and age, as well as the date of dispensing and the number of administration units. Drugs are classified according to the World Health Organization’s anatomical therapeutic chemical (ATC) classification system. Drugs administered in hospitals and nursing homes are not recorded.

Data on diagnoses from primary and secondary care were retrieved from the Norwegian Registry for Primary Health Care (NRPHC) and the Norwegian Patient Registry (NPR), respectively [25]. The NRPHC provides data on all contacts with primary care, and the diagnoses are recorded according to the International Classification of Primary Care, 2nd edition (ICPC-2). The NPR provides data on all contacts with secondary care, and diagnoses are recorded according to the International Classification of Diseases, 10th revision (ICD-10). Data on cancer diagnosis (ICD-10 codes C00–C97, D45–47) were retrieved from the Cancer Registry of Norway, which provides data on all incident malignancies [25]. Socioeconomic data were retrieved from population registers from Statistics Norway.

### Study drugs

Opioids included in the study were opioid analgesics (ATC code N02A). Opioids used in opioid maintenance treatment (N07BC) and antitussives (R05DA) were not included. Opioid analgesics are only available on prescription in Norway.

### Study population and definition of long-term opioid use

A detailed description of the study population can be found in Nestvold et al. [5] and the applied exclusions in Supplemental Figure 1. Persons included in our study population were those aged 18 years and above, who during the study period (2010–2019) became either long-term opioid users (cases) or short-term opioid users (controls). As previously described by Hamina et al. [26], a person had long-term opioid use if he/she: (a) had a first filled prescription of opioids and then a second within 91–180 days from the first one; and (b) were dispensed 90 or more administration units (e.g. tablets) of opioids within the first 90 days. This definition assumes daily opioid use for more than 3 months, which corresponds to the International Association for the Study of Pain/ICD-11 definition of chronic pain as pain that persists or recurs for more than 3 months [27]. A person could have several long-term periods, separated by intervals of more than 180 days without opioid prescriptions, but in the current study, only the first period of long-term use for cases was included, and each case was matched to four controls on sex, age and index year (the year during which they received the first opioid prescription in their first long-term use period). The final study cohort consisted of 215,642 cases (long-term opioid use) and 862,568 controls (short-term opioid use).

### Immigration variable definition

The two main exposure variables used in this study were: (a) immigration status as either immigrant or native-born; and (b) region of birth. Immigrants were defined as persons who have themselves immigrated to Norway, who were born abroad, and who have two foreign-born parents and four foreign-born grandparents in accordance with standards from Statistics Norway [28].

The variable ‘region of birth’ represents the resident country of the mother at the time of birth, with resident countries grouped into ‘regions’. Regions of birth were categorised as follows: Norway, European Union/European Economic Area (EU/EEA) outside of Norway, Europe outside of EU, Africa, Asia including Turkey, North America, Central and South America, and Oceania.

### Definition of covariates

We included several covariates related to long-term opioid use based on previous literature and clinical relevance [4, 5, 25, 26]. Socioeconomic variables were education, disposable household income, single-person household, living in a densely versus sparsely populated area, unemployment and receiving disability pension. All socioeconomic variables were measured one year before the index year or within one to 365 days before the index date, depending on the level of detail available for each variable (Supplemental Table I).

For the adjusted analysis, we also included the following pain-related diagnoses and symptoms: back pain, arthrosis, migraine and headaches, abdominal and pelvic pain, neuropathic pain, and other medically relevant covariates such as mental disorders (anxiety and depression), substance use disorders and sleep disturbance/disorders (Supplemental Table II). All these covariates were defined as having at least one diagnosis and/or symptom registered in primary or secondary care within one to 365 days before the index date. The use of benzodiazepines or benzodiazepine-related drugs (BZDRs) was defined as having at least one filled prescription of a BZDR within one to 365 days before the index date. The data do not include information on whether the pain-related diagnoses and symptoms included were the reasons for prescribing opioids long-term.

### Analytical strategy

Descriptive statistics are presented as proportions (*n*, %), medians with interquartile ranges (IQRs) and means with standard deviations (SDs). Both descriptive and analytical statistics were performed on three stratified age groups ‘18–44 years of age’, ‘45–67 years of age’ and ‘68 years of age and above’. Employment-related variables were not assessed among persons in the oldest age group as the Norwegian retirement age is 67 years. Separate logistic regression analyses were performed to investigate the relationship between: (a) immigration status and long-term opioid use; and (b) region of birth and long-term opioid use. Two different regression models were applied for both variables separately. Model 1 adjusted for socioeconomic factors, while model 2 (fully adjusted analyses) further adjusted for back pain, arthrosis, migraine, abdominal and pelvic pain, neuropathic pain, anxiety, depression, substance use disorders, sleep disturbance/disorders and the use of BZDRs.

We performed two supplemental analyses, first by excluding all persons with a previous cancer diagnosis, due to the link between cancer-related pain and the use of opioid analgesics, and because the prevalence of cancer types differs between immigrants and native-born people [29]. Secondarily, we performed an analysis stratified on sex, as differences between men and women on areas of mental health and socioeconomic status tend to be greater among immigrants than native-born people [11].

Missing values are reported in descriptive tables but were excluded from the regression analyses (complete case analysis). Stata 16.1 was used for statistical analyses (StataCorp, 2021).

## Results

### Characteristics of the study population

In the youngest age group (18–44 years), the proportion of immigrants was lower among the cases compared with the controls (15% vs. 18%) (Table I). No such difference was observed for the two older age groups. EU/EAA outside of Norway and Asia including Turkey were the most frequent regions of birth besides Norway among the cases in all three age groups (18–44 years: 5.5% vs. 6.8%; 45–67 years: 4.8% vs. 4.2%; 68 years and above: 2.7% vs. 0.8%) (Table I).

**Table I.** Immigration background for cases (long-term opioid users) and controls (short-term opioid users) stratified on age groups in the period 2010–2019 in Norway.

	18–44 Years (*N*=239,360)				45–67 Years (*N*=428,025)				68 Years and above (*N*=410,825)			
	Cases (*N*=47,872)		Controls (*N*=191,488)		Cases (*N*=85,605)		Controls (*N*=342,420)		Cases (*N*=82,165)		Controls (*N*=328,660)	
Age, mean (SD)	34.5	(7.0)	34.5	(7.0)	56.4	(6.6)	56.4	(6.6)	78.5	(7.0)	78.5	(7.0)
Women, *n* (%)	26,152	(54.6)	104,608	(54.6)	44,106	(51.5)	176,424	(51.5)	50,901	(62.0)	203,604	(62.0)
Immigrant status, *n* (%)												
Immigrant	7158	(15.0)	34751	(18.2)	9417	(11.0)	36,317	(11.0)	13,821	(4.2)	3392	(4.1)
Native	40,714	(85.1)	156,737	(81.9)	76,188	(89.0)	306,103	(89.4)	314,839	(95.8)	78,773	(95.9)
Regions of birth, *n* (%)												
Norway	39,791	(83.1)	153,039	(79.9)	75,095	(87.7)	301,862	(88.2)	78,585	(95.6)	314,045	(95.6)
EU/EEA	2653	(5.5)	14,542	(7.6)	4078	(4.8)	16,278	(4.8)	2255	(2.7)	9212	(2.8)
Europe outside of EU	761	(1.6)	3617	(1.9)	967	(1.1)	3663	(1.1)	232	(0.3)	857	(0.3)
Africa	781	(1.6)	4328	(2.3)	857	(1.0)	3575	(1.0)	137	(0.2)	587	(0.2)
Asia including Turkey	3255	(6.8)	12,752	(6.7)	3590	(4.2)	12,963	(3.8)	620	(0.8)	2466	(0.8)
North America	147	(0.3)	634	(0.3)	479	(0.6)	1721	(0.5)	239	(0.3)	1032	(0.3)
Central and South America	353	(0.7)	1873	(1.0)	428	(0.5)	1770	(0.5)	83	(0.1)	356	(0.1)
Oceania	28	(0.1)	153	(0.1)	32	(0.0)	134	(0.0)	13	(0.0)	68	(0.0)
Missing	103	(0.2)	550	(0.3)	79	(0.1)	454	(0.1)	1	(0.0)	37	(0.0)

SD: standard deviation; EU: European Union; EEA: European Economic Area.

Both immigrant and native cases in all three age groups had lower socioeconomic status when compared with their respective controls (Table II). Compared with native cases, immigrant cases on average had higher education (18–44 years: 21.4% vs. 19.7% in the highest education group; 45–67 years: 26.5% vs. 18.6%; 68 years and above: 23.4% vs. 12.8%), lower income (18–44 years: 42.3% vs. 27.4% in the lowest income group; 45–67 years: 36.9% vs. 17.0%; 68 years and above: 42.3% vs. 35.3%), higher unemployment rate (18–44 years: 37.1% vs. 35.0%; 45–67 years: 50.3% vs. 44.9%) but lower disability pension rate (18–44 years: 4.1% vs. 12.5%; 45–67 years: 23.2% vs. 40.4%). They also more often lived in more densely populated areas and in multi-person households.

**Table II.** Characteristics of cases (long-term opioid use) and controls (short-term opioid use) stratified on immigration status for three age groups: 18–44 years (*N*=239,340), 45–67 years (*N*=428,025) and 68 years and above (*N*=410,825).

Age 18–44 years	Immigrants (*N*=35,799)				Natives (*N*=203,561)			
	Immigrant cases (*N*=7158)		Controls (*N*=28,641)		Native cases (*N*=40,714)		Controls (*N*=162,847)	
Age, mean (SD)	35.9	(6.0)	35.9	(6.0)	34.2	(7.2)	34.2	(7.2)
Women, *n* %	3206	(44.8)	12,837	(44.8)	22,946	(56.4)	91,771	(56.4)
Education, *n* (%)								
No education	120	(1.7)	75	(0.3)	<5^a^	(0.0)	443	(0.3)
Lower secondary school	2708	(37.8)	6617	(23.1)	15,854	(38.9)	39,371	(24.2)
Upper secondary school	2085	(29.1)	11,521	(40.2)	16,443	(40.4)	64,316	(39.5)
Higher education	1533	(21.4)	9643	(33.7)	8011	(19.7)	54,238	(33.3)
Missing	712	(10.0)	785	(2.7)	<406^a^	(<1.0)	4479	(2.8)
Income quartile, *n* (%)								
0–25%	3031	(42.3)	5699	(19.9)	11,170	(27.4)	36,063	(22.2)
25–50%	2026	(28.3)	7205	(25.2)	11,775	(28.9)	41,567	(25.5)
50–75%	1176	(16.4)	7769	(27.1)	9994	(24.6)	43,123	(26.5)
75–100%	748	(10.5)	7740	(27.0)	7577	(18.6)	40,947	(25.1)
Missing	177	(2.5)	228	(0.8)	198	(0.5)	1138	(0.7)
Single person household, *n* (%)								
Yes	1358	(19.0)	5097	(17.8)	8616	(21.2)	28,869	(17.7)
No	5547	(77.5)	23,248	(81.2)	31,837	(78.2)	132,406	(81.3)
Missing	253	(3.5)	296	(1.0)	261	(0.6)	1572	(1.0)
Living in densely populated areas, *n* (%)								
Yes	6267	(87.6)	23,522	(82.1)	31,896	(78.3)	133,680	(82.1)
No	585	(8.2)	4752	(16.6)	8381	(20.6)	27,251	(16.7)
Missing	306	(4.3)	367	(1.3)	437	(1.1)	1916	(1.2)
Unemployed, *n* (%)								
Yes	2655	(37.1)	5122	(17.9)	14,248	(35.0)	31,687	(19.5)
No	4349	(60.8)	23,381	(81.6)	26,325	(64.7)	130,424	(80.1)
Missing	154	(2.2)	138	(0.5)	141	(0.4)	736	(0.5)
On disability pension, *n* (%)								
Yes	291	(4.1)	1413	(4.9)	5068	(12.5)	7390	(4.5)
No	6867	(95.9)	27,228	(95.1)	35,646	(87.6)	155,457	(95.5)
Depression, *n* (%)	1049	(14.7)	2580	(9.0)	6374	(15.7)	15,606	(9.6)
Anxiety, *n* (%)	561	(7.8)	1853	(6.5)	5273	(13.0)	10,802	(6.6)
Substance use disorder, *n* (%)	126	(1.8)	493	(1.7)	2633	(6.5)	2620	(1.6)
Back pain, *n* (%)	3092	(43.2)	5921	(20.7)	13,549	(33.3)	31,736	(19.5)
Neuropathic pain, *n* (%)	257	(3.6)	609	(2.1)	1456	(3.5)	3535	(2.2)
Arthrosis, *n* (%)	263	(3.7)	701	(2.5)	1416	(3.5)	394	(2.4)
Migraine, *n* (%)	877	(12.3)	2803	(9.8)	5247	(12.9)	16,741	(10.3)
Abdominal and pelvic pain, *n* (%)	1276	(17.8)	3747	(13.1)	6909	(17.0)	23,126	(14.2)
Sleep disturbance/disorders, *n* (%)	518	(7.2)	1827	(6.4)	4611	(11.3)	9916	(6.1)
BZDR, *n* (%)	1458	(20.4)	3388	(11.8)	12,678	(31.1)	18,363	(11.3)
Age 45–67 years	Immigrants (*N*=47,085)				Natives (*N*=380,940)			
	Immigrant cases (*N*=9417)		Controls (*N*=37,668)		Native cases (*N*=76,188)		Controls (*N*=304,752)	
Age, mean (SD)	54.3	(6.3)	54.3	(6.3)	56.6	(6.7)	56.6	(6.6)
Women, *n* %	4330	(46.0)	17,290	(45.9)	39,776	(52.2)	159,134	(52.2)
Education, *n* (%)								
No education	346	(3.7)	128	(0.3)	187	(0.3)	1221	(0.4)
Lower secondary school	2953	(31.4)	8966	(23.8)	24,613	(32.3)	71,312	(23.4)
Upper secondary school	3043	(32.3)	17,765	(47.2)	36,627	(48.1)	146,386	(48.0)
Higher education	2495	(26.5)	10,379	(27.6)	14,172	(18.6)	82,767	(27.2)
Missing	580	(6.2)	430	(1.1)	589	(0.8)	3066	(1.0)
Income quartile, *n* (%)								
0–25%	3479	(36.9)	4506	(12.0)	12,937	(17.0)	35,988	(11.8)
25–50%	2278	(24.2)	7155	(19.0)	18,419	(24.2)	59,539	(19.5)
50–75%	1878	(19.9)	9711	(25.8)	19,747	(25.9)	79,068	(26.0)
75–100%	1659	(17.6)	16,108	(42.8)	24,848	(32.6)	128,842	(42.3)
Missing	123	(1.3)	188	(0.5)	237	(0.3)	1315	(0.4)
Age 45–67 years	Immigrants (*N*=47,085)				Natives (*N*=380,940)			
	Immigrant cases (*N*=9417)		Controls (*N*=37,668)		Native cases (*N*=76,188)		Controls (*N*=304,752)	
Single person household, *n* (%)								
Yes	2017	(21.4)	6841	(18.2)	19,911	(26.1)	58,283	(19.1)
No	7242	(76.9)	30,614	(81.3)	55,973	(73.5)	244,957	(80.4)
Missing	158	(1.7)	213	(0.6)	304	(0.4)	1512	(0.5)
Living in densely populated areas, *n* (%)								
Yes	8267	(87.8)	29,565	(78.5)	58,010	(76.1)	239,161	(78.5)
No	932	(9.9)	7814	(20.7)	17,632	(23.1)	63,594	(20.9)
Missing	218	(2.3)	289	(0.8)	546	(0.7)	1997	(0.7)
Unemployed, *n* (%)								
Yes	4732	(50.3)	9142	(24.3)	34,184	(44.9)	88,886	(29.2)
No	4588	(48.7)	28,422	(75.5)	41,831	(54.9)	215,052	(70.6)
Missing	97	(1.0)	104	(0.3)	173	(0.2)	814	(0.3)
On disability pension, *n* (%)								
Yes	2183	(23.2)	6925	(18.4)	30,758	(40.4)	66,445	(21.8)
No	7234	(76.8)	30,743	(81.6)	45,430	(59.6)	238,307	(78.2)
Depression, *n* (%)	1149	(12.2)	2941	(7.8)	7949	(10.4)	23,684	(7.8)
Anxiety, *n* (%)	528	(5.6)	1769	(4.7)	5388	(7.1)	13,439	(4.4)
Substance use disorder, *n* (%)	85	(0.9)	234	(0.6)	1763	(2.3)	1606	(0.5)
Back pain, *n* (%)	3243	(34.4)	8176	(21.7)	20,555	(27.0)	65,193	(21.4)
Neuropathic pain, *n* (%)	461	(4.9)	1540	(4.0)	3681	(4.8)	13,295	(4.4)
Arthrosis, *n* (%)	1590	(16.9)	4767	(12.7)	11,758	(15.4)	46,070	(15.1)
Migraine, *n* (%)	824	(8.8)	2680	(7.1)	4766	(6.3)	20,903	(6.9)
Abdominal and pelvic pain, *n* (%)	1457	(15.5)	4464	(11.9)	9877	(13.0)	37,934	(12.5)
Sleep disturbance/disorders, *n* (%)	810	(8.6)	3656	(9.7)	10,263	(13.5)	30,331	(10.0)
BZDR, *n* (%)	2612	(27.7)	7467	(19.8)	31,557	(41.4)	67,901	(22.3)
Age 68 years and above	Immigrants (*N*=16,950)				Natives (*N*=393,875)			
	Immigrant cases (*N*=3392)		Controls (*N*=13,558)		Native cases (*N*=78,773)		Controls (*N*=315,102)	
Age, mean (SD)	77.1	(6.7)	77.1	(6.7	78.5	(7.0)	78.5	(7.0)
Women, *n* %	2088	(61.6)	8368	(61.7)	48,813	(62.0)	195,236	(62.0)
Education, *n* (%)								
No education	188	(5.5)	44	(0.3)	246	(0.3)	1132	(0.4)
Lower secondary school	995	(29.3)	4636	(34.2)	32,712	(41.5)	112,599	(35.7)
Upper secondary school	1233	(36.4)	6367	(47.0)	35,407	(45.0)	145,384	(46.1)
Higher education	793	(23.4)	2435	(18.0)	10,101	(12.8)	54,180	(17.2)
Missing	183	(5.4)	76	(0.6)	307	(0.4)	1807	(0.6)
Income quartile, *n* (%)								
0–25%	1433	(42.3)	3712	(27.4)	27,766	(35.3)	98,372	(31.2)
25–50%	940	(27.7)	4518	(33.3)	27,185	(34.5)	104,843	(33.3)
50–75%	585	(17.3)	3084	(22.8)	15,311	(19.4)	67,363	(21.4)
75–100%	395	(11.7)	2211	(16.3)	8435	(10.7)	43,906	(13.9)
Missing	39	(1.2)	33	(0.2)	76	(0.1)	618	(0.2)
Single person household, *n* (%)								
Yes	1378	(40.6)	5235	(38.6)	34,973	(44.4)	129,580	(41.1)
No	1974	(58.2)	8284	(61.1)	43,700	(55.5)	184,867	(58.7)
Missing	40	(1.2)	39	(0.3)	100	(0.1)	655	(0.2)
Living in densely populated areas, *n* (%)								
Yes	3018	(89.0)	10,742	(79.2)	61,747	(78.4)	249,982	(79.3)
No	330	(9.7)	2763	(20.4)	16,868	(21.4)	64,231	(20.4)
Missing	44	(1.3)	53	(0.4)	158	(0.2)	889	(0.3)
Unemployed, *n* (%)								
Yes	n/a	n/a	n/a	n/a	n/a	n/a	n/a	n/a
No	n/a	n/a	n/a	n/a	n/a	n/a	n/a	n/a
Missing	n/a	n/a	n/a	n/a	n/a	n/a	n/a	n/a
On disability pension, *n* (%)								
Yes	n/a	n/a	n/a	n/a	n/a	n/a	n/a	n/a
No	n/a	n/a	n/a	n/a	n/a	n/a	n/a	n/a
Age 68 years and above	Immigrants (*N*=16,950)				Natives (*N*=393,875)			
	Immigrant cases (*N*=3392)		Controls (*N*=13,558)		Native cases (*N*=78,773)		Controls (*N*=315,102)	
Depression, *n* (%)	176	(5.2)	746	(5.5)	4798	(6.1)	18,330	(5.8)
Anxiety, *n* (%)	85	(2.5)	409	(3.0)	2628	(3.3)	9315	(3.0)
Substance use disorder, *n* (%)	<5	(<0.2)	14	(0.1)	179	(0.2)	472	(0.2)
Back pain, *n* (%)	814	(24.0)	3205	(23.6)	21,025	(26.7)	75,680	(24.0)
Neuropathic pain, *n* (%)	140	(4.1)	818	(6.0)	3781	(4.8)	18,988	(6.0)
Arthrosis, *n* (%)	836	(24.7)	3720	(27.4)	16,967	(21.5)	85,560	(27.2)
Migraine, n (%)	127	(3.7)	654	(4.8)	2576	(3.3)	16,225	(5.2)
Abdominal and pelvic pain, *n* (%)	464	(13.7)	2209	(16.3)	10,012	(12.7)	49,963	(15.9)
Sleep disturbance/disorders, *n* (%)	370	(10.9)	1995	(14.7)	11,141	(14.1)	46,711	(14.8)
BZDR, *n* (%)	1412	(41.6)	5575	(41.1)	40,224	(51.1)	133,416	(42.3)

Cases and corresponding controls were matched on age, sex and index year but not on immigration status or country of origin. Immigrant-specific controls are therefore not necessarily immigrants.

SD: standard deviation; BZDR: benzodiazepines or benzodiazepine-related drugs.

a To avoid the reporting of exact numbers between one and four we have masked percentages as ⩽X% where X=4/n*100, and the masking of another column to make sure this exact number cannot be calculated from the rest.

In the youngest age group, immigrant cases had lower proportions of people with depression, anxiety, substance use disorders, migraine, sleep disturbance/disorders and the use of BZDRs, and higher proportions of people with neuropathic pain, arthrosis and abdominal and pelvic pain when compared with native cases.

### Association between immigration background and long-term opioid use

**Figure 1.**
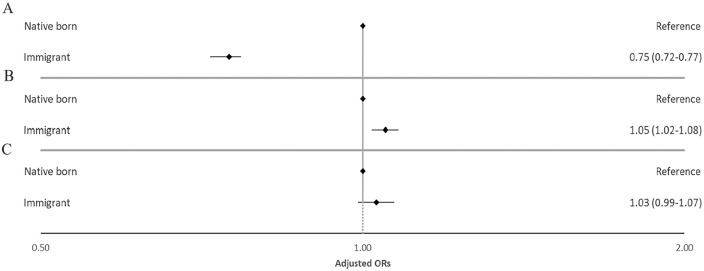
Adjusted odds ratios (aORs) with 95% confidence intervals (CIs) for the association between immigration status and long-term opioid use in Norway 2010–2019 stratified on three age groups (A: 18–44 years; B: 45–67 years; C: 68 years and above). The model adjusted for the highest achieved level of education, income quartile, living in a single person household, living in densely populated areas, being unemployed or receiving disability pension, arthrosis, migraine, abdominal and pelvic pain, back pain, neuropathic pain, depression, anxiety, substance use disorder, sleep disturbance/disorders and the use of benzodiazepines or benzodiazepine-related drugs. The *X* axis is shown as a logarithmic scale.

In the fully adjusted logistic regression analysis, immigrants in the youngest age group (18–44 years) had lower odds of long-term opioid use when compared with natives (aOR 0.75; 95% CI [0.72–0.77]), whereas immigrants in the middle age group (44–67 years) had slightly higher odds (aOR 1.05; [1.02–1.08]) (Figure 1, Table III). No association was observed for the oldest age group (68 years and above). Low income, low education, unemployment and receiving disability pension were all inversely associated with becoming a long-term opioid user in the fully adjusted model (Supplemental Table III). Of the different co-morbidities used for adjustment, the use of BZDRs had the strongest association with the outcome in all three age groups. Of the previously diagnosed pain-related conditions, back pain had the strongest association in the younger age group, while abdominal and pelvic pain had the strongest association for the two older age groups. Substance use disorders were also associated with long-term opioid use in the youngest age group, but not in the two older age groups (Supplemental Table III).

**Table III.** Unadjusted odds ratio (OR) and adjusted odds ratios (aORs) with 95% confidence intervals (CIs) for the association between immigration status and long-term opioid use, stratified by age groups (18–44 years, 45–67 years and 68 years and above).

	18–44 Years (*N*=239,360)					
	Unadjusted (*N*=239,360)		Model 1 (*N*=230,832)		Model 2 (*N*=230,832)	
	OR	95% CI	aOR	95% CI	aOR	95% CI
Native	Ref	–	Ref	–	Ref	–
Immigrant	0.79	0.77–0.82	0.75	0.72–0.77	0.75	0.72–0.77
	45–67 Years (*N*=428,025)					
	Unadjusted (*N*=428,205)		Model 1 (*N*=420,811)		Model 2 (*N*=420,811)	
	OR	95% CI	aOR	95% CI	aOR	95% CI
Native	Ref	–	Ref	–	Ref	–
Immigrant	1.04	1.02–1.07	0.99	0.96–1.02	1.05	1.02–1.08
	68 Years and above (*N*=410,825)					
	Unadjusted (*N*=410,825)		Model 1 (*N*=407,443)		Model 2 (*N*=407,443)	
	OR	95% CI	aOR	95% CI	aOR	95% CI
Native	Ref	–	Ref	–	Ref	–
Immigrant	0.98	0.94–1.02	0.99	0.95–1.03	1.03	0.99–1.07

Model 1 adjusted for socioeconomic status variables; highest achieved level of education, income quartile, living in a single person household, living in densely populated areas, being unemployed or receiving disability pension.

Model 2 further adjusted for clinically relevant comorbidities; arthrosis, migraine, abdominal and pelvic pain, neuropathic pain, depression, anxiety, substance use disorder, sleep disturbance/disorders and the use of benzodiazepines or benzodiazepine-related drugs.

‘Immigrant’ is here defined as being born outside of Norway to two foreign-born parents and four foreign-born grandparents.

Ref: reference category.

**Figure 2.**
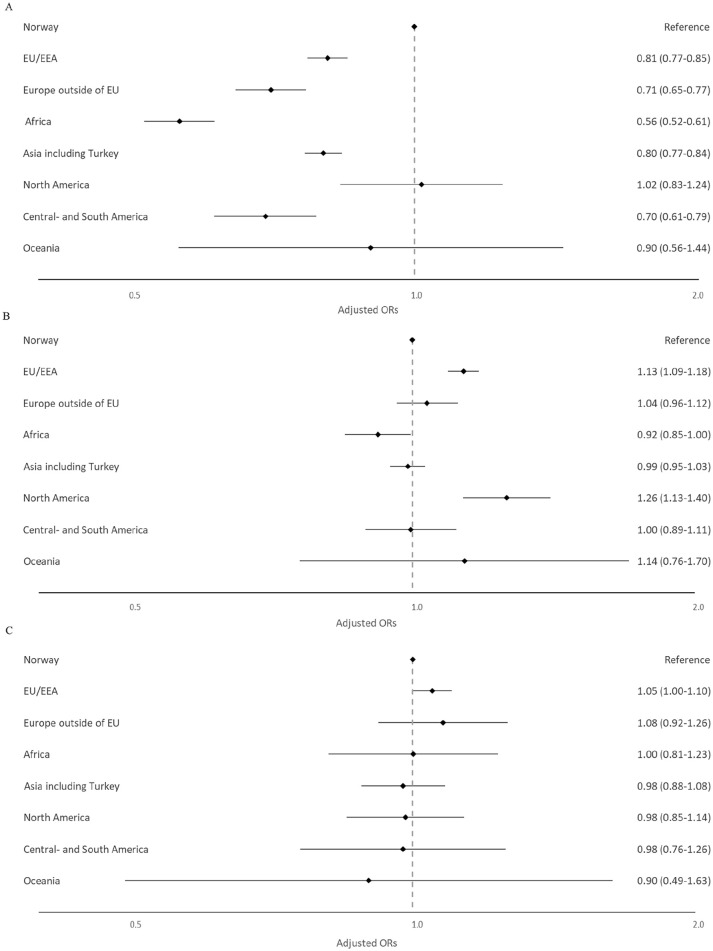
Adjusted odds ratios (aORs) with 95% confidence intervals (CIs) for the association between region of birth and long-term opioid use in Norway 2010–2019 stratified on three age groups (A: 18–44 years; B: 45–67 years; C: 68 years and above). The model adjusted for the highest achieved level of education, income quartile, living in a single person household, living in densely populated areas, being unemployed or receiving disability pension, arthrosis, migraine, abdominal and pelvic pain, back pain, neuropathic pain, depression, anxiety, substance use disorder, sleep disturbance/disorders and the use of benzodiazepines or benzodiazepine-related drugs. The *X* axis is shown as a logarithmic scale.

When compared with Norway, lower odds of long-term opioid use were observed for all regions of birth in the youngest age group except for North America and Oceania, with Africa and Central and South America showing the strongest association (Africa: aOR 0.56 [0.52–0.61]; Central and South America: aOR 0.70 [0.61–0.79]) (Figure 2, Table IV). For the middle age group, higher odds were observed among those being born in North America (aOR 1.26 [1.13–1.40]) and the EU/EEA outside of Norway (aOR 1.13 [1.09–1.18]). No associations between regions of birth and long-term opioid use were observed for the oldest age group.

**Table IV.** Unadjusted odds ratio (OR) and adjusted odds ratios (aORs) with 95% confidence intervals (CIs) for the association between region of birth and long-term opioid use, stratified by age groups (18–44 years, 45–67 years and 68 years and above).

	18–44 Years (*N*=239,360)					
	OR	95% CI	aOR	95% CI	aOR	95% CI
Region of birth	Unadjusted (*N*=238,707)		Model 1 (*N*=230,832)		Model 2 (*N*=230,832)	
Norway	Ref	–	Ref	–	Ref	–
EU/EEA	0.70	0.67–0.73	0.79	0.76–0.83	0.81	0.77–0.85
Europe outside of EU	0.81	0.75–0.88	0.75	0.69–0.82	0.71	0.65–0.77
Africa	0.69	0.64–0.75	0.54	0.50–0.59	0.56	0.52–0.61
Asia including Turkey	0.98	0.94–1.02	0.82	0.78–0.85	0.80	0.77–0.84
North America	0.89	0.75–1.07	1.06	0.87–1.28	1.02	0.84–1.24
Central and South America	0.72	0.65–0.81	0.71	0.63–0.80	0.70	0.61–0.79
Oceania	0.70	0.47–1.05	0.78	0.49–1.23	0.90	0.56–1.44
	45–67 Years (*N*=428,025)					
	Unadjusted (*N*=427,492)		Model 1 (*N*=420,811)		Model 2 (*N*=420,811)	
Norway	Ref	–	Ref	–	Ref	–
EU/EEA	1.01	0.97–1.04	1.10	1.06–1.14	1.13	1.09–1.18
Europe outside of EU	1.06	0.99–1.14	0.99	0.92–1.07	1.04	0.96–1.12
Africa	0.96	0.89–1.04	0.83	0.77–0.90	0.92	0.85–1.00
Asia including Turkey	1.11	1.07–1.16	0.92	0.88–0.96	0.99	0.95–1.03
North America	1.12	1.01–1.24	1.27	1.14–1.41	1.26	1.13–1.40
Central and South America	0.97	0.87–1.08	0.97	0.87–1.08	1.00	0.89–1.11
Oceania	0.96	0.65–1.41	1.14	0.76–1.70	1.14	0.76–1.70
	68 Years and above (*N*=410,825)					
	Unadjusted (*N*=410,787)		Model 1 (*N*=407,443)		Model 2 (*N*=407,443)	
Norway	Ref	–	Ref	–	Ref	–
EU/EEA	0.98	0.93–1.03	1.03	0.98–1.08	1.05	1.00–1.10
Europe outside of EU	1.08	0.94–1.25	1.01	0.86–1.12	1.08	0.92–1.26
Africa	0.93	0.77–1.12	0.89	0.72–1.09	1.00	0.81–1.23
Asia including Turkey	1.00	0.92–1.10	0.89	0.80–0.98	0.98	0.88–1.08
North America	0.93	0.80–1.07	0.99	0.86–1.14	0.98	0.85–1.14
Central and South America	0.93	0.73–1.18	0.89	0.70–1.15	0.98	0.76–1.26
Oceania	0.76	0.42–1.38	0.84	0.46–1.52	0.90	0.49–1.63

Model 1 adjusted for socioeconomic status variables; highest achieved level of education, income quartile, living in a single person household, living in densely populated areas, being unemployed or receiving disability pension.

Model 2 further adjusted for arthrosis, migraine, abdominal and pelvic pain, neuropathic pain, depression, anxiety, substance use disorder, sleep disturbance/disorders and the use of benzodiazepines or benzodiazepine-related drugs.

Ref: reference category.

Supplemental analyses when excluding those with previous cancer diagnoses showed similar results to our main analyses, although somewhat attenuated (Supplemental Tables IV and V).

Supplemental analyses stratified on sex showed that female immigrants in the youngest age group had lower odds of developing long-term opioid use than male immigrants. For the two older age groups, no association was found among female immigrants whereas male immigrants had higher odds of long-term opioid use. For both sexes, young immigrants from Africa had the lowest odds of long-term opioid use. In the two oldest age groups, male immigrants from the EU/EEA had the highest odds of long-term opioid use (Supplemental Tables VI–IX).

## Discussion

We found that young immigrants in Norway had lower odds of developing long-term opioid use when compared with native-born people, whereas middle-aged immigrants had slightly increased odds, and no association was found for the oldest age group. The strength of the association varied for immigrants born in different regions: young African immigrants had the lowest and middle-aged immigrants from EU/EEA and North America had the highest odds of developing long-term use compared with native-born people. Young immigrants with long-term opioid use had lower proportions of diagnosed anxiety, substance use disorders, sleep disturbance/disorders and the use of BZDRs, but higher prevalence of pain-related diagnoses when compared with native-born people with long-term opioid use.

Our findings on the youngest group are in line with previous studies that have suggested that prescription opioid use is less prevalent among immigrants in general, and that immigrants have a lower risk of illicit opioid use and of substance use disorders [11, 16, 30]. Our results for the middle age group differ from those found in a previous Norwegian study by Svendsen et al. [4], who suggested that middle-aged immigrants had a lower risk of developing persistent opioid use. This could be explained by differences in study design, age stratification, definitions of long-term opioid use and immigrant status, earlier study period and lack of clinical covariate adjustment.

Although previous studies have shown higher levels of self-reported back pain, neck pain and chronic pain among immigrants [12, 13, 24], adjusting for clinically diagnosed pain disorders did not change the estimates of association between long-term opioid use and immigration status in the youngest age group. It is important to note, however, that there might be a discrepancy between clinical diagnoses of pain disorders and self-reported pain among immigrants. However, it is interesting to see how the reduced risk of long-term opioid use persists so strongly through our adjusted analyses, suggesting that the effect could be more due to cultural differences in this age group than economic differences.

Immigrants tend to approach the population average on most health-related aspects, including medicine use and health literacy as time goes on [16, 23, 30], with health literacy being defined in Norway as an individual’s ability to acquire, understand, assess and utilise the health system to promote and benefit their health [31]. Although we did not have data on how long the immigrants had been in Norway, this phenomenon might partially explain why we do not see the same results from the middle age group. We found that middle-aged immigrants, especially those from North America and the EU/EEA were more likely to develop long-term opioid use than native-born people. It seems logical that individuals from regions with high levels of opioid use, such as North America and parts of EU/EEA, are more likely to develop long-term use also as immigrants.

Those who prescribe opioids might do so differently for immigrants compared with native-born people, but as both immigrants and natives received opioids at least once, this suggests that our finding is not fully due to lack of interaction with the healthcare system or access to analgesics. While barriers to entry are somewhat removed by the study design, there may be cultural or language barriers that make continued use of the healthcare system less pleasant for immigrants [19, 21]. Previous studies also report perceptions of discrimination as an impeding factor for continued contact with healthcare professionals [21, 22]. Also, more common among immigrants is a lack of trust in the healthcare system compared with native-born people [32]. These factors may explain why immigrants had a less frequent long-term use of opioid analgesics compared with native-born people despite having a higher prevalence of pain-related diagnoses, especially back pain, and lower socioeconomic status.

Supplemental analysis also discovered that removing individuals with cancer from the analyses only slightly attenuated the results, which further emphasises the impact of immigration on the risk of developing long-term opioid use on its own. Supplemental sex-stratified analysis did however show that young immigrant women had lower odds than young immigrant men, which was most exemplified for young female immigrants from Africa.

As the number of immigrants will continue to increase internationally in the future, these findings show that the healthcare system needs to facilitate treatment that takes cultural background into account to ensure proper pain treatment.

### Strengths and limitations

The major strength of this study was that all individuals who filled at least one prescription for opioids in Norway between 2010 and 2019 were included. We were also able to control for many relevant covariates, including socioeconomic status and clinically relevant comorbidities due to the completeness and level of detail available in the nationwide registers.

One limitation of this study is that we did not have data on when immigration occurred. Younger immigrants in our dataset therefore had a higher chance of showing a ‘recency effect’ and were more affected by differences in culture and language than older immigrants. Another limitation is the lack of detail in our ‘region of birth’ variable. As we only have main regions/continents as categories available, finding meaningful explanations from cultural differences is difficult and can only result in broad generalisations. Another limitation was the lack of pre-immigration background information such as refugee status, past traumatic events, or previous drug use before 2010, which are not considered in this study.

## Conclusions

In conclusion, young immigrants are less likely, middle-aged immigrants more likely, and older immigrants as likely to develop long-term opioid use compared with native-born Norwegians. This effect varies between regions of birth and may be explained by cultural differences, as it persists when adjusting for socioeconomic status, pain-related conditions, mental disorders and the use of BZDRs. Future studies should further investigate the nuances of different countries of birth, their associations with long-term opioid use and the possible cultural or religious reasons for differences in long-term opioid use.

## Supplemental Material

sj-docx-1-sjp-10.1177_14034948241266744 – Supplemental material for The risk of long-term opioid use among immigrants: a national registry-linkage study Supplemental material, sj-docx-1-sjp-10.1177_14034948241266744 for The risk of long-term opioid use among immigrants: a national registry-linkage study by Håkon H. Nestvold, Svetlana Skurtveit, Aleksi Hamina, Vidar Hjellvik and Ingvild Odsbu in Scandinavian Journal of Public Health
