# Supplementary material for: The risk of long-term opioid use among immigrants: a national registry-linkage study
Source: Scand J Public Health. 2024 Aug 22;53(7):764–75. doi: 10.1177/14034948241266744 (PMC12598078; doi:10.1177/14034948241266744)

**Supplementary**

**Supplementary Table 1.** Socioeconomic variable categories used for this study.

| Variable name | Operationalisation | Assessment window |
| --- | --- | --- |
| Education | Highest achieved education level (i) no education, (ii) lower secondary school, (iii) upper secondary school, (iv) higher education. | Assessed the year before index year. |
| Income | Quartiles of available income within the study cohort: (i) 0-25%, (ii) 25-50%, (iii) 50-75%, (iv) 75-100% | Assessed the year before index year. |
| Single person household | Yes/no | Assessed the year before index year. |
| Rural living | Yes/no | Assessed the year before index year. |
| Unemployed | Yes/no | Assessed the year before index year. |
| Disability pension | Yes/no | Assessed from –365 to -1 day before index date. |

**Supplementary Table 2.** Definition of comorbidities and other medication use assessed from 365 to 1 day before index date.

| Variable name | Primary care, ICPC-2 codes | Secondary care, ICD-10 codes | ATC codes |
| --- | --- | --- | --- |
| Back pain | L02, L03, L84, L86 | M54 |  |
| Arthritis | L89-91 | M15-19 |  |
| Migraine and headaches | N01, N89, N90, N95 | G43-44, R51 |  |
| Abdominal and pelvic pain | D01, D02, D06 | R10 |  |
| Neuropathic pain | N92, N94 | G50-G59, G60-G64 |  |
| Depression | P76 | F32-34, F39 |  |
| Anxiety | P74 | F40-44 |  |
| Substance abuse/mental and behavioral disorders due to psychoactive substance use (Substance use disorder) | P18, P19 | F11-F19 (excluding F17) |  |
| Sleep disturbance/disorders | P06 | F51, G47 |  |
| Benzodiazepines/benzodiazepine-related drugs (BZDRs) |  | | N05BA, N05CD, N05CF, N03AE01 |

**Abbreviations**: ICD-10. International Classification of Diseases. version 10; ICPC-2. International Classification of Primary Care. 2nd edition. ATC: Anatomical Therapeutic Chemical classification system.

**Supplementary Table 3.** Extended table of adjusted odds ratios (aORs) for the association between being an immigrant and developing long-term opioid use, and for socioeconomic risk factors and clinical comorbidities used in adjustment models.

| Regression model 2 | Age 18-44 years | | Age 45-67 years | | Age 68 years and above | |
| --- | --- | --- | --- | --- | --- | --- |
|  | aOR | 95% CI | aOR | 95% CI | aOR | 95% CI |
| Being an immigrant | 0.75 | 0.72 -0.77 | 1.05 | 1.02 - 1.08 | 1.03 | 0.99 - 1.07 |
| No | 1 | ~ | 1 | ~ | 1 | ~ |
| Education, n (%) |  |  |  |  |  |  |
| No education | 1.23 | 1.00 - 1.51 | 1.38 | 1.24 - 1.54 | 1.82 | 1.62 - 2.04 |
| Lower secondary school | 1.86 | 1.81 - 1.92 | 1.36 | 1.33 - 1.39 | 1.33 | 1.30 - 1.37 |
| Upper secondary school | 1.40 | 1.36 - 1.44 | 1.16 | 1.14 - 1.19 | 1.17 | 1.14 - 1.20 |
| Higher education | 1 | ~ | 1 | ~ | 1 | ~ |
| Income, median (iqr) |  |  |  |  |  |  |
| 0-25% | 1.24 | 1.20 - 1.29 | 1.32 | 1.28 - 1.35 | 1.23 | 1.19 - 1.26 |
| 25-50% | 1.28 | 1.24 - 1.32 | 1.21 | 1.18 - 1.23 | 1.19 | 1.16 - 1.23 |
| 50-75% | 1.15 | 1.12 - 1.19 | 1.14 | 1.11 - 1.16 | 1.12 | 1.09 - 1.15 |
| 75-100% | 1 | ~ | 1 | ~ | 1 | ~ |
| Single person household | 1.03 | 1.00 - 1.05 | 1.11 | 1.09 - 1.13 | 1.03 | 1.01 - 1.05 |
| Dense living | 0.92 | 0.89 - 0.95 | 0.94 | 0.92 - 0.96 | 0.97 | 0.95 - 0.99 |
| Unemployment | 1.57 | 1.53 - 1.61 | 1.30 | 1.28 - 1.33 | n/a | ~ |
| On disability pension | 1.26 | 1.21 - 1.32 | 1.53 | 1.50 - 1.56 | n/a | ~ |
| Comorbidities |  |  |  |  |  |  |
| Depression | 1.06 | 1.03 - 1.10 | 1.37 | 1.35 - 1.40 | 1.17 | 1.14 - 1.19 |
| Anxiety | 0.99 | 0.96 - 1.03 | 1.02 | 1.00 - 1.05 | 0.75 | 0.74 - 0.77 |
| Substance use disorder | 1.58 | 1.49 - 1.68 | 0.85 | 0.83 - 0.88 | 0.62 | 0.60 - 0.65 |
| Neuropathic pain | 1.37 | 1.29 - 1.46 | 0.9 | 0.88 - 0.92 | 0.75 | 0.73 - 0.76 |
| Back pain | 2.10 | 2.05 - 2.15 | 0.98 | 0.95 - 1.02 | 0.81 | 0.78 - 0.84 |
| Arthrosis | 1.42 | 1.34 - 1.51 | 0.96 | 0.94 - 0.99 | 0.96 | 0.93 - 0.99 |
| Migraine and headaches | 1.13 | 1.09 - 1.17 | 0.94 | 0.91 - 0.97 | 0.99 | 0.95 - 1.04 |
| Abdominal and pelvic pain | 1.05 | 1.02 - 1.08 | 1.94 | 1.81 - 2.08 | 1.43 | 1.20 - 1.70 |
| Sleep disturbance | 0.98 | 0.94 - 1.02 | 0.87 | 0.85 - 0.90 | 0.78 | 0.76 - 0.80 |
| BZDR | 2.56 | 2.49 - 2.64 | 2.03 | 1.99 - 2.06 | 1.51 | 1.49 - 1.54 |

**Note:** Model 2 adjusted for all variables used in the table; highest achieved level of education, income quartile, living in a single person household, living in densely populated areas, being unemployed or receiving disability pension; arthrosis, migraine and headaches, abdominal and pelvic pain, neuropathic pain, depression, anxiety, substance use disorder, sleep disturbance/disorders, and use of benzodiazepines or benzodiazepine-related drugs (BZDR). “immigrant" here defined as being born outside of Norway to two foreign-born parents and four foreign-born grandparents.

**Supplementary Table 4.** Sensitivity analysis for those without a previous diagnosis of cancer (ICD10: C00-C97, D45-47)**.** Unadjusted odds ratio (OR) and adjusted odds ratios (aORs) with 95% confidence intervals (CI) for the association between immigration status and long-term opioid use, stratified by age groups (18-44 years, 45-67 years, and 68 years and above).

|  | **18-44 years (N = 235 658)** | | | | | |
| --- | --- | --- | --- | --- | --- | --- |
|  | Unadjusted  (N = 235 658) | | Model 1  (N = 227 223) | | Model 2  (N = 227 223) | |
|  | OR | 95% CI | aOR | 95% CI | aOR | 95% CI |
| Native | Ref | - | Ref | - | Ref | - |
| Immigrant | 0.79 | 0.77 - 0.81 | 0.74 | 0.72 - 0.77 | 0.74 | 0.72 - 0.77 |
|  | **45-67 years (N = 399 057)** | | | | | |
|  | Unadjusted  (N = 399 057) | | Model 1  (N = 392 167) | | Model 2  (N = 392 167) | |
|  | OR | 95% CI | aOR | 95% CI | aOR | 95% CI |
| Native | Ref | - | Ref | - | Ref | - |
| Immigrant | 1.07 | 1.04 - 1.10 | 1.01 | 0.98 - 1.04 | 1.07 | 1.04 - 1.10 |
|  | **68 years and above (N = 357 421)** | | | | | |
|  | Unadjusted  (N = 357 421) | | Model 1  (N = 354 404) | | Model 2  (N = 354 404) | |
|  | OR | 95% CI | aOR | 95% CI | aOR | 95% CI |
| Native | Ref | - | Ref | - | Ref | - |
| Immigrant | 0.98 | 0.94 - 1.02 | 0.99 | 0.95 - 1.03 | 1.03 | 0.98 - 1.08 |

**Note:** Model 1 adjusted for socioeconomic status variables; highest achieved level of education, income quartile, living in a single person household, living in densely populated areas, being unemployed or receiving disability pension. Model 2 further adjusted for clinically relevant comorbidities; arthrosis, migraine, abdominal and pelvic pain, neuropathic pain, depression, anxiety, substance use disorder, sleep disturbance/disorders, and use of benzodiazepines or benzodiazepine-related drugs. “immigrant" here defined as being born outside of Norway to two foreign-born parents and four foreign-born grandparents. Ref, Reference category.

**Supplementary Table 5.** Sensitivity analysis for those without a previous diagnosis of cancer**.** Unadjusted odds ratio (OR) and adjusted odds ratios (aORs) with 95% confidence intervals (CI) for the association between region of birth and long-term opioid use, stratified by age groups (18-44 years, 45-67 years, and 68 years and above).

|  | **18-44 years (N = 235 658)** | | | | | |
| --- | --- | --- | --- | --- | --- | --- |
|  | OR | 95% CI | aOR | 95% CI | aOR | 95% CI |
| **Region of birth** | Unadjusted (N = 235 005) | | Model 1 (N = 227 223) | | Model 2 (N = 227 223) | |
| Norway | Ref | ~ ~ | Ref | ~ ~ | Ref | ~ ~ |
| EU/EEA | 0.70 | 0.67 - 0.73 | 0.79 | 0.75 - 0.83 | 0.80 | 0.76 - 0.84 |
| Europe outside of EU | 0.81 | 0.75 - 0.88 | 0.75 | 0.69 - 0.81 | 0.70 | 0.64 - 0.77 |
| Africa | 0.69 | 0.64 - 0.74 | 0.53 | 0.49 - 0.58 | 0.56 | 0.51 - 0.61 |
| Asia including Turkey | 0.98 | 0.94 - 1.02 | 0.82 | 0.78 - 0.85 | 0.80 | 0.76 - 0.84 |
| North America | 0.87 | 0.73 - 1.05 | 1.04 | 0.85 - 1.26 | 1.00 | 0.81 - 1.22 |
| Central- and South America | 0.73 | 0.65 - 0.82 | 0.71 | 0.63 - 0.81 | 0.70 | 0.62 - 0.80 |
| Oceania | 0.67 | 0.44 - 1.02 | 0.73 | 0.45 - 1.17 | 0.84 | 0.52 - 1.40 |
|  | **45-67 years (N = 399 057)** | | | | | |
|  | Unadjusted (N = 398 524) | | Model 1 (N = 392 167) | | Model 2 (N = 392 167) | |
| Norway | Ref | ~ ~ | Ref | ~ ~ | Ref | ~ ~ |
| EU/EEA | 1.03 | 0.99 - 1.07 | 1.12 | 1.08 - 1.17 | 1.15 | 1.11 - 1.20 |
| Europe outside of EU | 1.07 | 0.99 - 1.15 | 0.99 | 0.92 - 1.08 | 1.04 | 0.96 - 1.13 |
| Africa | 0.99 | 0.93 - 1.08 | 0.86 | 0.79 - 0.93 | 0.94 | 0.87 - 1.03 |
| Asia including Turkey | 1.16 | 1.11 - 1.20 | 0.95 | 0.91 - 0.99 | 1.02 | 0.97 - 1.06 |
| North America | 1.12 | 1.01 - 1.25 | 1.27 | 1.14 - 1.42 | 1.26 | 1.12 - 1.41 |
| Central- and South America | 1.00 | 0.90 - 1.12 | 0.99 | 0.88 - 1.11 | 1.02 | 0.91 - 1.15 |
| Oceania | 0.98 | 0.65 -1.46 | 1.16 | 0.77 - 1.76 | 1.16 | 0.76 - 1.77 |
|  | **68 years and above (N = 357 421)** | | | | | |
|  | Unadjusted (N = 357 383) | | Model 1 (N = 354 404) | | Model 2 (N = 354 404) | |
| Norway | Ref | ~ ~ | Ref | ~ ~ | Ref | ~ ~ |
| EU/EEA | 0.99 | 0.94 - 1.04 | 1.04 | 0.99 - 1.10 | 1.06 | 1.01 - 1.12 |
| Europe outside of EU | 1.13 | 0.97 - 1.33 | 1.06 | 0.89 - 1.25 | 1.13 | 0.95 - 1.35 |
| Africa | 0.89 | 0.73 - 1.10 | 0.84 | 0.66 - 1.06 | 0.96 | 0.76 - 1.21 |
| Asia including Turkey | 1.00 | 0.91 - 1.10 | 0.87 | 0.78 - 0.97 | 0.96 | 0.86 - 1.07 |
| North America | 0.88 | 0.75 - 1.03 | 0.94 | 0.80 - 1.11 | 0.93 | 0.79 - 1.10 |
| Central- and South America | 0.87 | 0.67 - 1.14 | 0.84 | 0.64 - 1.12 | 0.92 | 0.70 - 1.23 |
| Oceania | 0.63 | 0.31 - 1.26 | 0.69 | 0.34 - 1.39 | 0.74 | 0.37 - 1.50 |

**Note:** Model 1 adjusted for socioeconomic status variables; highest achieved level of education, income quartile, living in a single person household, living in densely populated areas, being unemployed or receiving disability pension. Model 2 further adjusted for; arthrosis, migraine, abdominal and pelvic pain, neuropathic pain, depression, anxiety, substance use disorder, sleep disturbance/disorders, and use of benzodiazepines or benzodiazepine-related drugs. Ref, Reference category.

**Supplementary table 6.** Sensitivity analysis for females only showing the association between long-term opioid use and immigration status. Unadjusted odds ratio (OR) and adjusted odds ratios (aORs) with 95% confidence intervals (CI) for the association between immigration status and long-term opioid use, stratified by age groups (18-44 years, 45-67 years, and 68 years and above).

|  | **18-44 years (N = 130 760)** | | | | | |
| --- | --- | --- | --- | --- | --- | --- |
|  | Unadjusted | | Model 1 | | Model 2 | |
|  | (N = 130 760) | | (N = 127 134) | | (N = 127 134) | |
|  | OR | 95% CI | aOR | 95% CI | aOR | 95% CI |
| Native | Ref | - | Ref | - | Ref | - |
| Immigrant | 0.66 | 0.63 - 0.68 | 0.62 | 0.59 - 0.65 | 0.62 | 0.60 - 0.65 |
|  | **45-67 years (N = 220 530)** | | | | | |
|  | Unadjusted | | Model 1 | | Model 2 | |
|  | (N = 220 530) | | (N = 217 572) | | (N = 217 572) | |
|  | OR | 95% CI | aOR | 95% CI | aOR | 95% CI |
| Native | Ref | - | Ref | - | Ref | - |
| Immigrant | 0.95 | 0.92 - 0.98 | 0.92 | 0.89 - 0.96 | 0.99 | 0.96 - 1.03 |
|  | **68 years and above (N = 254 505)** | | | | | |
|  | Unadjusted | | Model 1 | | Model 2 | |
|  | (N = 254 505) | | (N = 252 538) | | (N = 252 538) | |
|  | OR | 95% CI | aOR | 95% CI | aOR | 95% CI |
| Native | Ref | - | Ref | - | Ref | - |
| Immigrant | 0.93 | 0.89 - 0.98 | 0.95 | 0.90 - 1.00 | 0.99 | 0.94 - 1.05 |

**Note:** Model 1 adjusted for socioeconomic status variables; highest achieved level of education, income quartile, living in a single person household, living in densely populated areas, being unemployed or receiving disability pension. Model 2 further adjusted for clinically relevant comorbidities; arthrosis, migraine, abdominal and pelvic pain, neuropathic pain, depression, anxiety, substance use disorder, sleep disturbance/disorders, and use of benzodiazepines or benzodiazepine-related drugs. “immigrant" here defined as being born outside of Norway to two foreign-born parents and four foreign-born grandparents. Ref, Reference category.

**Supplementary Table 7**. Sensitivity analysis for females only showing unadjusted odds ratio (OR) and adjusted odds ratios (aORs) with 95% confidence intervals (CI) for the association between region of birth and long-term opioid use, stratified by age groups (18-44 years, 45-67 years, and 68 years and above).

|  | **18-44 years (N = 130 629)** | | | | | |
| --- | --- | --- | --- | --- | --- | --- |
|  | OR | 95% CI | aOR | 95% CI | aOR | 95% CI |
| **Region of birth** | Unadjusted (N = 130 629) | | Model 1 (N =127 134) | | Model 2 (N =127 134) | |
| Norway | Ref | ~ ~ | Ref | ~ ~ | Ref | ~ ~ |
| EU/EEA | 0.68 | 0.64 - 0.72 | 0.76 | 0.71 - 0.82 | 0.77 | 0.72 - 0.93 |
| Europe outside of EU | 0.64 | 0.57 - 0.72 | 0.62 | 0.55 - 0.69 | 0.60 | 0.53 - 0.67 |
| Africa | 0.56 | 0.50 - 0.63 | 0.43 | 0.38 - 0.49 | 0.45 | 0.40 - 0.51 |
| Asia including Turkey | 0.74 | 0.70 - 0.79 | 0.62 | 0.59 - 0.67 | 0.62 | 0.58 - 0.66 |
| North America | 0.80 | 0.63 - 1.03 | 0.94 | 0.72 - 1.22 | 0.93 | 0.71 - 1.21 |
| Central- and South America | 0.69 | 0.60 - 0.79 | 0.67 | 0.58 - 0.78 | 0.67 | 0.58 - 0.79 |
| Oceania | 0.78 | 0.41 - 1.49 | 0.99 | 0.50 - 1.99 | 1.21 | 0.60 - 2.45 |
|  | **45-67 years (N = 220 382)** | | | | | |
|  | Unadjusted (N = 220 382) | | Model 1 (N = 217 572) | | Model 2 (N = 217 572) | |
| Norway | Ref | ~ ~ | Ref | ~ ~ | Ref | ~ ~ |
| EU/EEA | 0.95 | 0.90 - 1.00 | 1.06 | 1.00 - 1.12 | 1.08 | 1.02 - 1.15 |
| Europe outside of EU | 0.99 | 0.90 - 1.09 | 0.97 | 0.88 - 1.07 | 1.04 | 0.94 - 1.15 |
| Africa | 0.92 | 0.82 - 1.03 | 0.80 | 0.71 - 0.91 | 0.93 | 0.82 - 1.05 |
| Asia including Turkey | 0.96 | 0.91 - 1.02 | 0.81 | 0.76 - 0.86 | 0.90 | 0.85 - 0-96 |
| North America | 1.19 | 1.04 - 1.37 | 1.37 | 1.19 - 1.58 | 1.37 | 1.18 - 1.58 |
| Central- and South America | 0.95 | 0.83 - 1.09 | 0.96 | 0.83 - 1.11 | 1.01 | 0.88 - 1.17 |
| Oceania | 1.19 | 0.69 - 2.04 | 1.45 | 0.83 - 2.52 | 1.50 | 0.86 - 2.64 |
|  | **68 years and above (N = 254 448)** | | | | | |
|  | Unadjusted (N = 254 448) | | Model 1 (N = 252 538) | | Model 2 (N = 252 538) | |
| Norway | Ref | ~ ~ | Ref | ~ ~ | Ref | ~ ~ |
| EU/EEA | 0.92 | 0.87 - 0.98 | 0.98 | 0.92 - 1.04 | 1.00 | 0.94 - 1.06 |
| Europe outside of EU | 1.14 | 0.96 - 1.36 | 1.07 | 0.89 - 1.29 | 1.18 | 0.97 - 1.42 |
| Africa | 0.71 | 0.54 - 0.93 | 0.66 | 0.48 - 0.92 | 0.77 | 0.56 - 1.07 |
| Asia including Turkey | 0.92 | 0.82 - 1.04 | 0.77 | 0.97 - 0.92 | 0.87 | 0.75 - 1.01 |
| North America | 0.97 | 0.82 - 1.15 | 1.05 | 0.88 - 1.25 | 1.05 | 0.88 - 1.25 |
| Central- and South America | 0.92 | 0.69 - 1.24 | 0.89 | 0.65 - 1.20 | 1.00 | 0.73 - 1.37 |
| Oceania | 0.47 | 0.20 - 1.09 | 0.51 | 0.22 - 1.20 | 0.55 | 0.23 - 1.28 |

**Note:** Model 1 adjusted for socioeconomic status variables; highest achieved level of education, income quartile, living in a single person household, living in densely populated areas, being unemployed or receiving disability pension. Model 2 further adjusted for; arthrosis, migraine, abdominal and pelvic pain, neuropathic pain, depression, anxiety, substance use disorder, sleep disturbance/disorders, and use of benzodiazepines or benzodiazepine-related drugs. Ref, Reference category.

**Supplementary Table 8.** Sensitivity analysis for males only showing the association between long-term opioid use and immigration status. Unadjusted odds ratio (OR) and adjusted odds ratios (aORs) with 95% confidence intervals (CI) for the association between immigration status and long-term opioid use, stratified by age groups (18-44 years, 45-67 years, and 68 years and above).

|  | **18-44 years (N = 108 600)** | | | | | |
| --- | --- | --- | --- | --- | --- | --- |
|  | Unadjusted | | Model 1 | | Model 2 | |
|  | (N = 108 600) | | (N = 103 698) | | (N = 103 698) | |
|  | OR | 95% CI | aOR | 95% CI | aOR | 95% CI |
| Native | Ref | - | Ref | - | Ref | - |
| Immigrant | 0.96 | 0.92 - 0.99 | 0.91 | 0.87 - 0.95 | 0.89 | 0.85 - 0.93 |
|  | **45-67 years (N = 207 495)** | | | | | |
|  | Unadjusted | | Model 1 | | Model 2 | |
|  | (N = 207 495) | | (N = 203 239) | | (N = 203 239) | |
|  | OR | 95% CI | aOR | 95% CI | aOR | 95% CI |
| Native | Ref | - | Ref | - | Ref | - |
| Immigrant | 1.14 | 1.10 - 1.17 | 1.05 | 1.01 - 1.08 | 1.08 | 1.04 - 1.12 |
|  | **68 years and above (N = 156 320)** | | | | | |
|  | Unadjusted | | Model 1 | | Model 2 | |
|  | (N = 156 320) | | (N = 154 905) | | (N = 154 905) | |
|  | OR | 95% CI | aOR | 95% CI | aOR | 95% CI |
| Native | Ref | - | Ref | - | Ref | - |
| Immigrant | 1.07 | 1.01 - 1.14 | 1.05 | 0.99 - 1.12 | 1.09 | 1.02 - 1.16 |

**Note:** Model 1 adjusted for socioeconomic status variables; highest achieved level of education, income quartile, living in a single person household, living in densely populated areas, being unemployed or receiving disability pension. Model 2 further adjusted for clinically relevant comorbidities; arthrosis, migraine, abdominal and pelvic pain, neuropathic pain, depression, anxiety, substance use disorder, sleep disturbance/disorders, and use of benzodiazepines or benzodiazepine-related drugs. “immigrant" here defined as being born outside of Norway to two foreign-born parents and four foreign-born grandparents. Ref, Reference category.

**Supplementary table 9.** sensitivity analysis for males only showing unadjusted odds ratio (OR) and adjusted odds ratios (aORs) with 95% confidence intervals (CI) for the association between region of birth and long-term opioid use, stratified by age groups (18-44 years, 45-67 years, and 68 years and above).

|  | **18-44 years (N = 108 078)** | | | | | |
| --- | --- | --- | --- | --- | --- | --- |
|  | OR | 95% CI | aOR | 95% CI | aOR | 95% CI |
| **Region of birth** | Unadjusted (N = 108 078) | | Model 1 (N = 103 698) | | Model 2 (N = 103 698) | |
| Norway | Ref | ~ ~ | Ref | ~ ~ | Ref | ~ ~ |
| EU/EEA | 0.73 | 0.69 - 0.77 | 0.83 | 0.78 - 0.89 | 0.85 | 0.79 - 0.91 |
| Europe outside of EU | 1.08 | 0.96 - 1.21 | 0.94 | 0.84 - 1.07 | 0.85 | 0.75 - 0.97 |
| Africa | 0.87 | 0.78 - 0.97 | 0.69 | 0.61 - 0.77 | 0.71 | 0.63 - 0.80 |
| Asia including Turkey | 1.30 | 1.23 - 1.38 | 1.08 | 1.02 - 1.15 | 1.03 | 0.97 - 1.10 |
| North America | 1.01 | 0.78 - 1.32 | 1.23 | 0.93 - 1.64 | 1.14 | 0.85 - 1.53 |
| Central- and South America | 0.79 | 0.65 - 0.95 | 0.77 | 0.92 - 0.94 | 0.74 | 0.60 - 0.92 |
| Oceania | 0.67 | 0.40 - 1.13 | 0.68 | 0.37 - 1.26 | 0.74 | 0.40 - 1.40 |
|  | **45-67 years (N = 207 110)** | | | | | |
|  | Unadjusted (N = 207 110) | | Model 1 (N = 203 239) | | Model 2 (N = 203 239) | |
| Norway | Ref | ~ ~ | Ref | ~ ~ | Ref | ~ ~ |
| EU/EEA | 1.06 | 1.01 - 1.11 | 1.12 | 1.06 - 1.18 | 1.16 | 1.10 - 1.22 |
| Europe outside of EU | 1.16 | 1.04 - 1.30 | 1.03 | 0.92 - 1.15 | 1.04 | 0.93 - 1.16 |
| Africa | 1.00 | 0.91 - 1.11 | 0.84 | 0.75 - 0.94 | 0.88 | 0.79 - 0.98 |
| Asia including Turkey | 1.29 | 1.23 - 1.36 | 1.03 | 0.97 - 1.09 | 1.05 | 0.99 - 1.12 |
| North America | 1.04 | 0.89 - 1.21 | 1.16 | 0.99 - 1.36 | 1.14 | 0.97 - 1.34 |
| Central- and South America | 1.01 | 0.85 - 1.19 | 0.99 | 0.84 - 1.18 | 0.98 | 0.82 - 1.16 |
| Oceania | 0.79 | 0.45 - 1.37 | 0.89 | 0.50 - 1.59 | 0.84 | 0.46 - 1.50 |
|  | **68 years and above (N = 156 299)** | | | | | |
|  | Unadjusted (N = 156 299) | | Model 1 (N = 154 905) | | Model 2 (N = 154 905) | |
| Norway | Ref | ~ ~ | Ref | ~ ~ | Ref | ~ ~ |
| EU/EEA | 1.09 | 1.01 - 1.17 | 1.11 | 1.03 - 1.20 | 1.14 | 1.05 - 1.23 |
| Europe outside of EU | 0.95 | 0.72 - 1.24 | 0.88 | 0.66 - 1.17 | 0.90 | 0.68 - 1.20 |
| Africa | 1.24 | 0.96 - 1.60 | 1.12 | 0.85 - 1.47 | 1.21 | 0.92 - 1.60 |
| Asia including Turkey | 1.12 | 0.99 - 1.28 | 1.03 | 0.89 - 1.18 | 1.10 | 0.95 - 1.27 |
| North America | 0.84 | 0.65 - 1.07 | 0.87 | 0.68 - 1.13 | 0.85 | 0.66 - 1.10 |
| Central- and South America | 0.95 | 0.63 - 1.44 | 0.91 | 0.59 - 1.41 | 0.93 | 0.60 - 1.43 |
| Oceania | 1.65 | 0.69 - 3.98 | 1.89 | 0.78 - 4.56 | 2.03 | 0.83 - 4.95 |

**Note:** Model 1 adjusted for socioeconomic status variables; highest achieved level of education, income quartile, living in a single person household, living in densely populated areas, being unemployed or receiving disability pension. Model 2 further adjusted for; arthrosis, migraine, abdominal and pelvic pain, neuropathic pain, depression, anxiety, substance use disorder, sleep disturbance/disorders, and use of benzodiazepines or benzodiazepine-related drugs. Ref, Reference category.

**Supplementary Figure 1.** Flowchart of selection of study population.


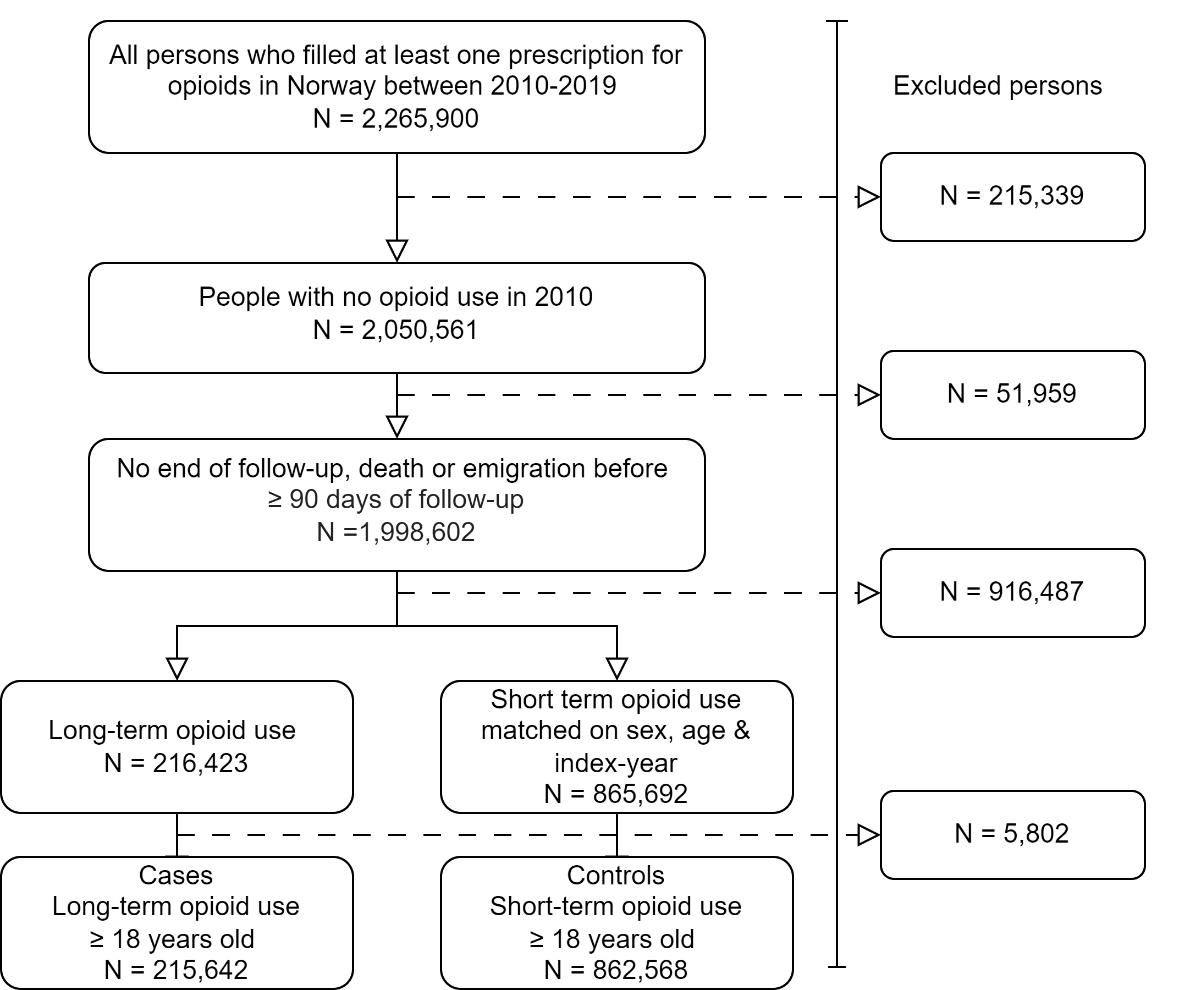

Supplement: sj-docx-1-sjp-10.1177_14034948241266744 – Supplemental material for The risk of long-term opioid use among immigrants: a national registry-linkage study [file sj-docx-1-sjp-10.1177_14034948241266744.docx]
